# Supplementary material for: α-Synuclein oligomers form by secondary nucleation
Source: Nat Commun. 2024 Aug 17;15:7083. doi: 10.1038/s41467-024-50692-4 (PMC11330488; doi:10.1038/s41467-024-50692-4)
Supplement: Supplementary file 1 — Supplementary Information [file 41467_2024_50692_MOESM1_ESM.pdf]

α-Synuclein oligomers form by secondary nucleation

Catherine K Xu[1,2], Georg Meisl[1], Ewa A Andrzejewska[1], Georg Krainer[1,3], Alexander J Dear[1,4], Marta Castellana Cruz[1], Soma Turi[1], Irina A Edu[1], Giorgio Vivacqua[5,6], Raphaël PB Jacquat[1], William E Arter[1], Maria Grazia Spillantini[6], Michele Vendruscolo[1], Sara Linse[4], Tuomas PJ Knowles[1,7]*

**To whom correspondence should be addressed: tpjk2@cam.ac.uk*

[1]Centre for Misfolding Diseases, Yusuf Hamied Department of Chemistry, University of Cambridge, Cambridge, UK

[2]Max Planck Institute for the Science of Light, Erlangen, Germany

[3] Institute of Molecular Biosciences (IMB), University of Graz, Graz, Austria

[4]Biochemistry and Structural Biology, Lund University, Lund, Sweden

[5]Integrated Research Center (PRAAB), Campus Biomedico University of Rome, Italy

[6]Department of Clinical Neurosciences, University of Cambridge, Cambridge, UK

[7]Cavendish Laboratory, University of Cambridge, Cambridge, UK

Supplementary methods

Analysis of μFFE data

Extraction of molecule concentrations and brightness from photon count data generally requires only one or a small number of molecules to simultaneously be located within the confocal spot and emitting photons. Such analysis approaches are therefore not suitable for our data, in which multiple protein species are always inside the confocal spot volume simultaneously. We therefore developed a theoretical framework which characterises the relationship between the detected distribution of photon count rates, and the brightness of the fluorophore. Our model comprises a confocal spot whose laser intensity is distributed as a 3-dimensional Gaussian function. Given that our data are acquired under laminar flow within a microfluidic

device, and that the laser dimensions are approximately 10-fold smaller than the channel dimensions, our model entails that fluorophores are randomly distributed in the xy plane, and move in the z (flow) direction at the same speed, with no deviation in xy position (i.e. no diffusion on this timescale).

By integrating the total laser intensity that would be experienced at every possible xy position, we thus determined that the probability that a given fluorophore emits a total of N^* photons while passing through the confocal volume is:

$$P_N(N = N^*) = \int_{I_{min}}^{I_{max}} \frac{e^{-alphaI}(\alpha I)^{N^*}}{IN^*!} dI \tag{1}$$

$$P_N(N = N^*) = \frac{\gamma(N^*, \alpha I_{min}) - \gamma(N^*, \alpha I_{max})}{N^*!} \tag{2}$$

where I_{min} and I_{max} are the minimum and maximum relative intensities (¡1), respectively, of the laser regions through which the fluorophore is allowed to travel, and α is the expected number of total photons emitted when the fluorophore path goes through the centre of the laser. The probability that two non-interacting fluorophores which simultaneously flow through the confocal volume emit a total of S^* photons is thus given by the convolution:

$$P_{S,2}(S = S^*) = (P_N * P_N)(N = N^*) \tag{3}$$

Similarly, the probability of S^* total photons emitted from n fluorophores can be determined by:

$$P_{S,n}(S = S^*) = (P_N * P_{S,n-1})(N = N^*) \tag{4}$$

With the assumption that the number of molecules simultaneously passing through the confocal volume is Poisson-distributed, the probability of detecting D^* photons is:

$$P_D(D = D^*) = \sum_{n=0}^{\infty} \frac{\lambda^{M^*} e^{-\lambda}}{M^*!} P_{S,n}(S = D^*) \tag{5}$$

where λ is the mean number of molecules simultaneously located within the confocal volume. From plotting the expected distributions of photon counts arising from fluorophores at different concentrations and with different brightnesses, it is clear that these two parameters cannot compensate for each other (Figure S1) both parameters can therefore theoretically be determined from experimental data.

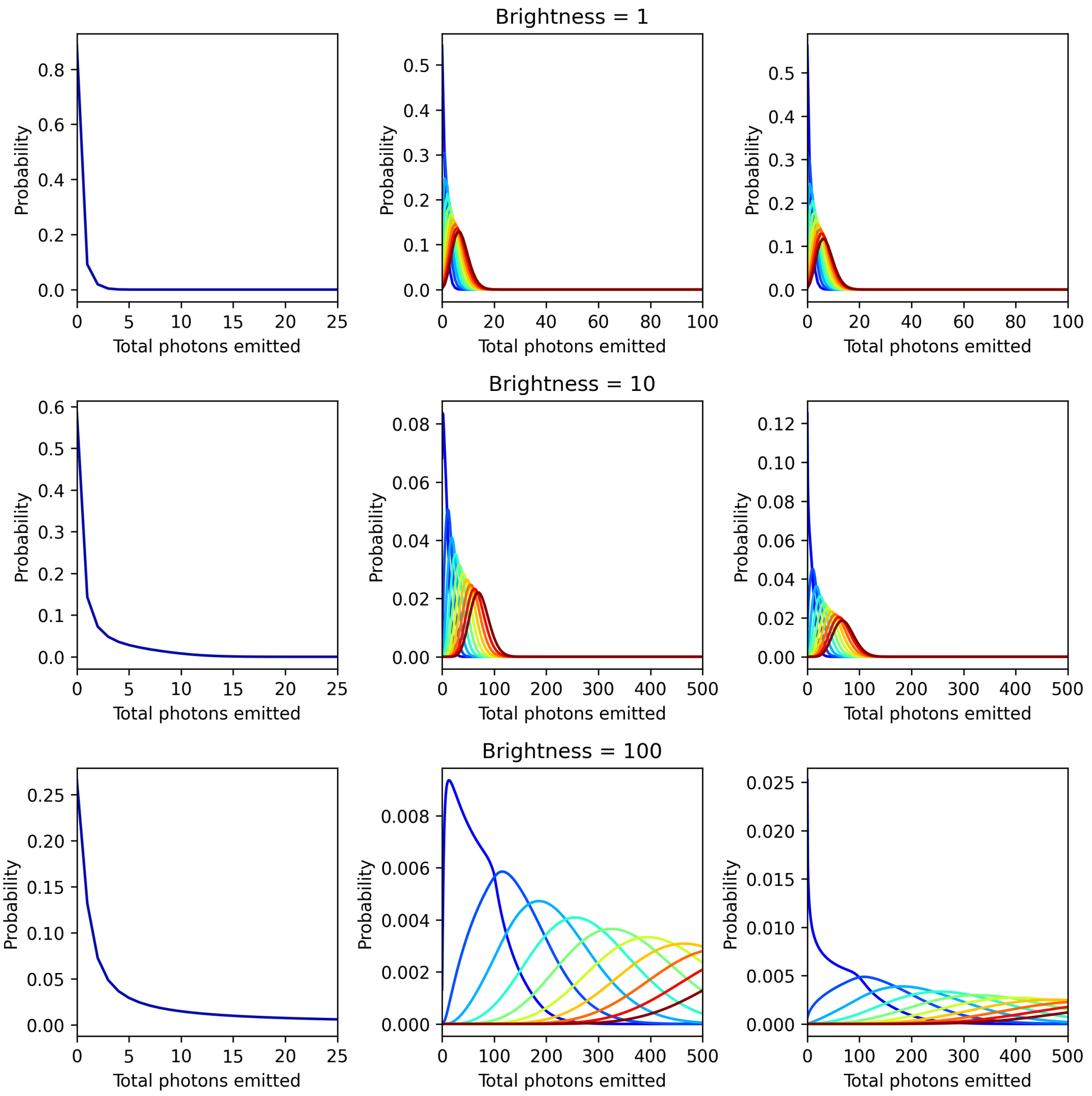

Figure S1: Expected photon count distributions of fluorophores passing through the confocal volume. Model distributions are shown for three different fluorophore brightnesses (α values of 1, 10, and 100). The expected distribution of emitted photons for a single fluorophore traversing the confocal volume (left) is shown alongside the expected distributions of multiple (5, 10, ... , 50 from blue to red) fluorophores being simultaneously located within the spot (middle). The right-hand plots show the expected distributions of emitted photons when multiple fluorophores pass through the confocal volume, with a mean of (5, 10, ... , 50 from blue to red) simultaneously inside the volume.

We fitted this model to data of AlexaFluor-488-labelled α-synuclein monomers (N122C variant) flowing through a straight microfluidic channel (height 50 µm, width 200 µm) at different concentrations across three orders of magnitude, with free parameters of concentration and brightness (α) (Figure S2). In all cases, the fitted α value was around 22 photons, and the relative concentrations differed by the expected factor of ten (Figure S3). We note that the absolute concentration fitted is dependent on the dimensions of the confocal spot, which is not well defined; the values we took to be approximately the standard deviations of the laser intensity were determined by fluorescence correlation spectroscopy [1].

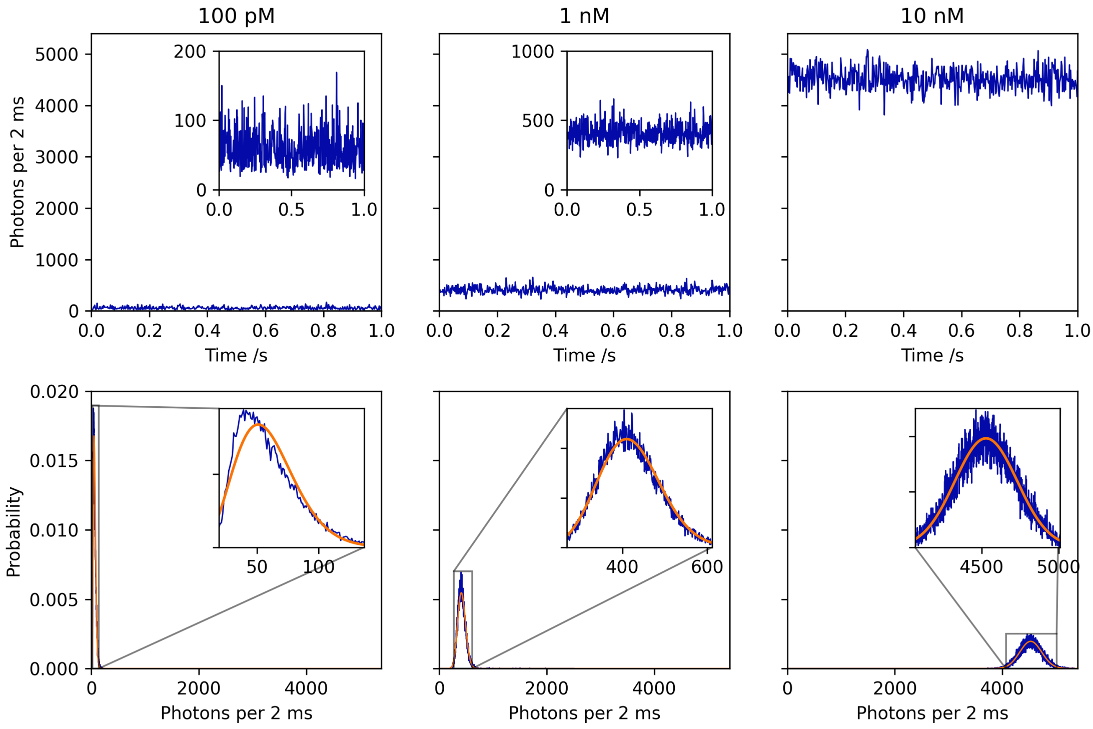

Figure S2: Determination of monomer brightness. Photon count data were acquired for monomeric α-synuclein at three different concentrations flowing through a straight microfluidic channel (height 50 µm, width 200 µm). Example sections of the timetraces are shown in the top row, and their corresponding photon count distributions (blue) shown alongside the fitted distributions (orange) based on our model (Equation 5) in the bottom row.

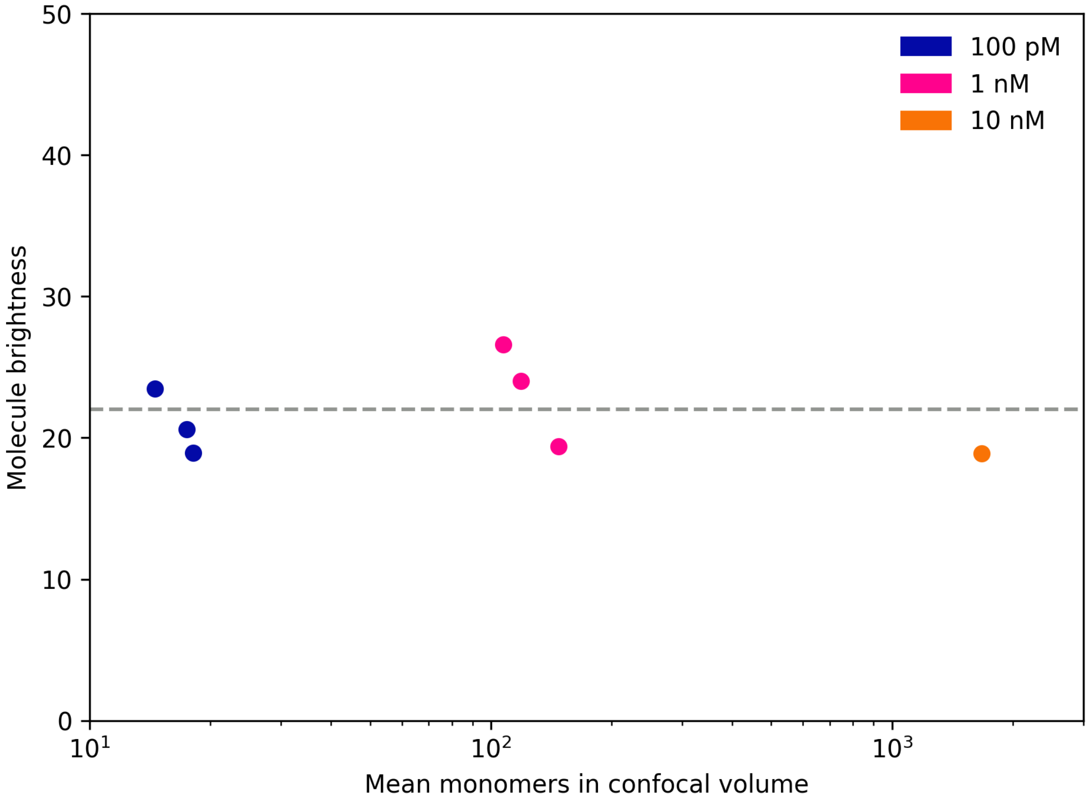

Figure S3: Fitted monomer parameters are consistent with experimental data. The fitted concentrations and brightnesses of labelled α-synuclein monomers in different measurement recordings are coloured according to the experimental concentrations. Both parameters are consistent across all data, validating our model. Based on these fits, the brightness value of monomers was taken to be 22 under our experimental conditions, as indicated by the grey dotted line.

Having determined the brightness of labelled monomeric α-synuclein, we next sought to determine the concentrations of oligomers from the photon count data. Oligomers contain multiple monomers and therefore multiple dyes, giving rise to a higher number of emitted photons per molecule; here, we estimate oligomer mass concentrations by assuming that dye molecules within oligomers contribute approximately linearly to the total photons detected. In order to avoid potential artefacts introduced by flow or electric field instabilities, and to account for the background signal from monomers, the rolling median (50 ms window) was subtracted from the timetraces, before applying thresholds on the photon count rate

to estimate the oligomer concentration (Figure S4).

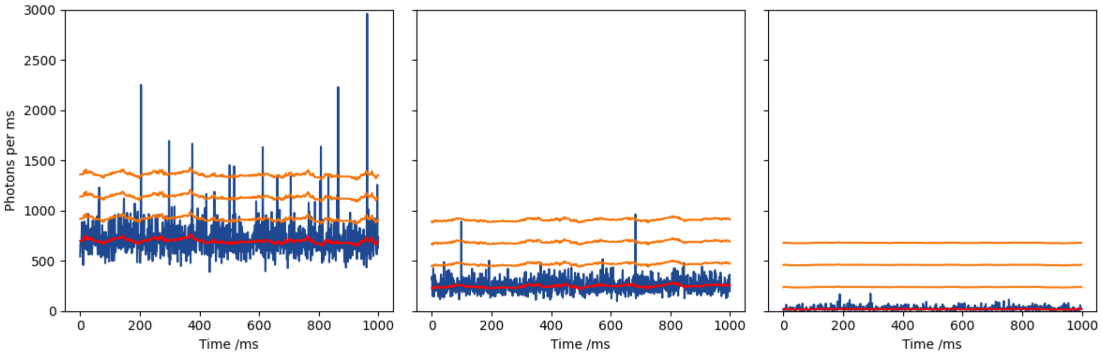

Figure S4: Example timetraces (blue) from µFFE are shown alongside the rolling median (red) and oligomer thresholds (orange) of 220, 440, and 660 photons/ms, corresponding to 10-mers, 20-mers, and 30-mers, respectively. Example timetrace sections are shown with different oligomer contents.

However, this approach systematically underestimates the oligomer concentration in the mixture. Using our model of photon count distributions detailed above, we calculated the fraction of oligomers which would be detected by using different photon count thresholds, for a range of oligomer sizes (Figure S5). Although the fraction detected is dependent on the size of the oligomers and threshold (440 photons in main text figure 4), the actual concentration of oligomers present in the aggregation mixture is likely to be at least tens to hundreds of fold higher than the apparent concentrations detected. However, the relative concentrations of oligomers between timepoints and measurements are preserved.

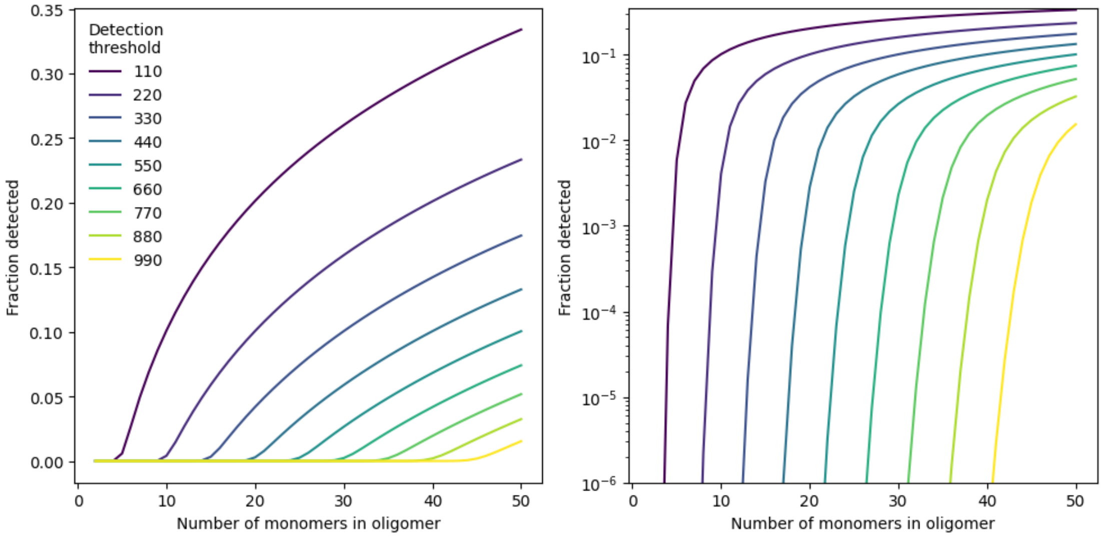

Figure S5: Fractions of oligomers that would be detected using different photon count thresholds. The same data are shown with both a linear (left) and logarithmic (right) y-scale.

In order to mitigate against variations in desalting efficiency and potential loss of protein by sticking to tubing, device, and/or syringe surfaces, the fraction of oligomer present in each timetrace was determined by comparing how many photon counts above the threshold compared to the total photon count. This fraction was then used in conjunction with the known supernatant concentration (Figure S7) to obtain relative oligomer mass concentrations throughout the aggregation reaction. This analysis was carried out for the whole channel width, to avoid exclusion of oligomers based on different electrophoretic mobilities.

Supplementary figures

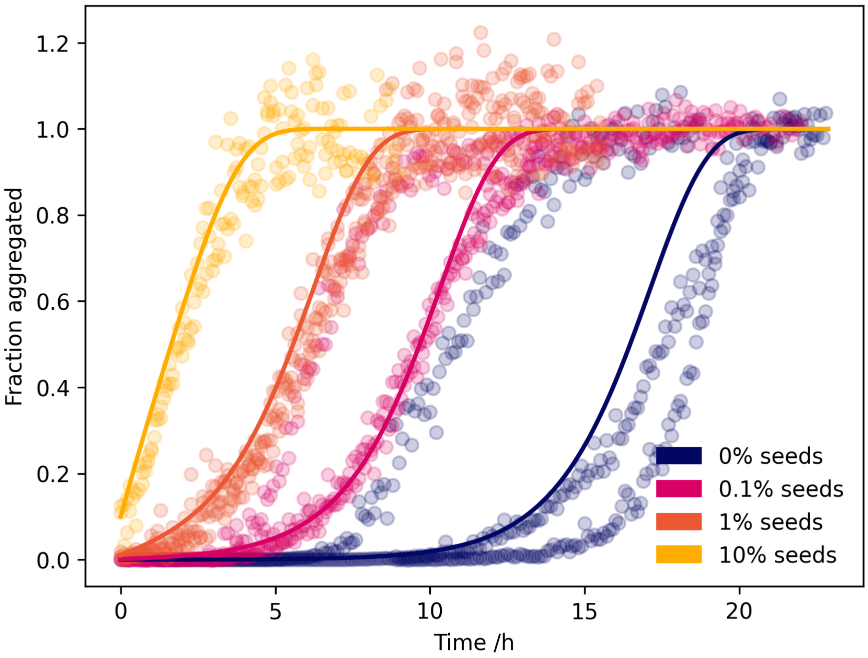

Figure S6: Labelling does not affect α-synuclein aggregation kinetics. Aggregation kinetics for unlabelled WT α-synuclein (100 µM total protein concentration) in the absence and presence of varying concentrations of seed fibrils, followed by thioflavin T fluorescence. Experimental data are shown as points, and fits as lines, with fitted parameters given in Table S1.

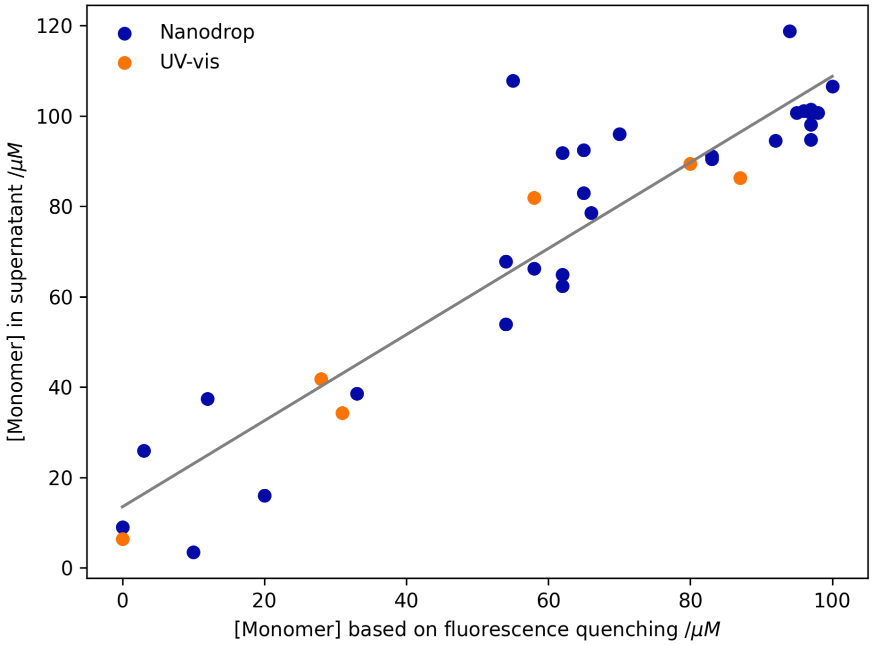

Figure S7: Aggregation of labelled α-synuclein can be followed by fluorescence quenching of the AlexaFluor-488 dye. The concentration of labelled α-synuclein the supernatant (21,130 rcf, 10 min) was determined by Nanodrop (blue) or UV-visible spectroscopy in a 1 cm-path length cuvette (orange) according to the absorbance at 495 nm using a molar extinction coefficient of 72,000 M^{-1} cm^{-1}. The supernatant concentrations were compared to the estimated non-fibrillar concentration by fluorescence quenching measured in the platereader, assuming a linear relationship between fluorescence and aggregated α-synuclein concentration. The soluble α-synuclein concentrations from these complementary methods are in good agreeement, with the gradient of the line of best fit being 0.95, with an R^2 value of 0.93.

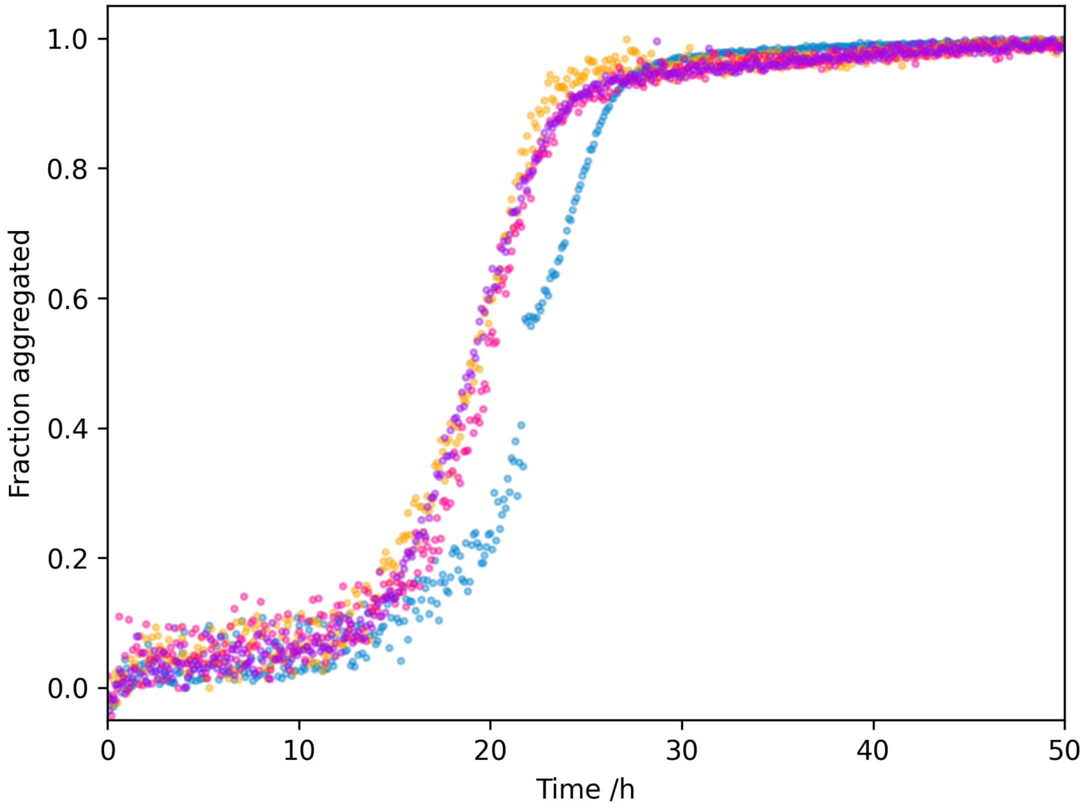

Figure S8: Example fibril formation kinetics of unseeded aggregation reactions of AlexaFluor-488-labelled N122C α-synuclein from different purification batches, shown as different colours.

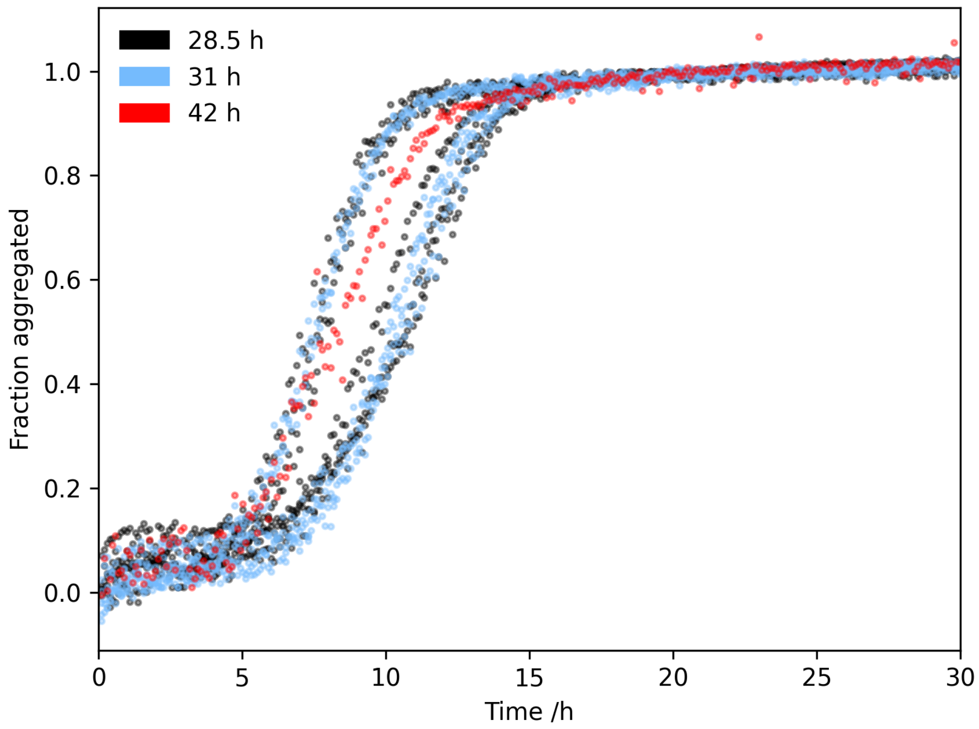

Figure S9: Seed fibril age has no effect on seeding propensity. Seed fibrils were extracted from unseeded aggregation reactions at 28, 31.5, and 42 hours after the reaction start, and used to seed further aggregation reactions (100 µM total protein concentration, with 1 µM seed fibrils (monomer equivalents)).

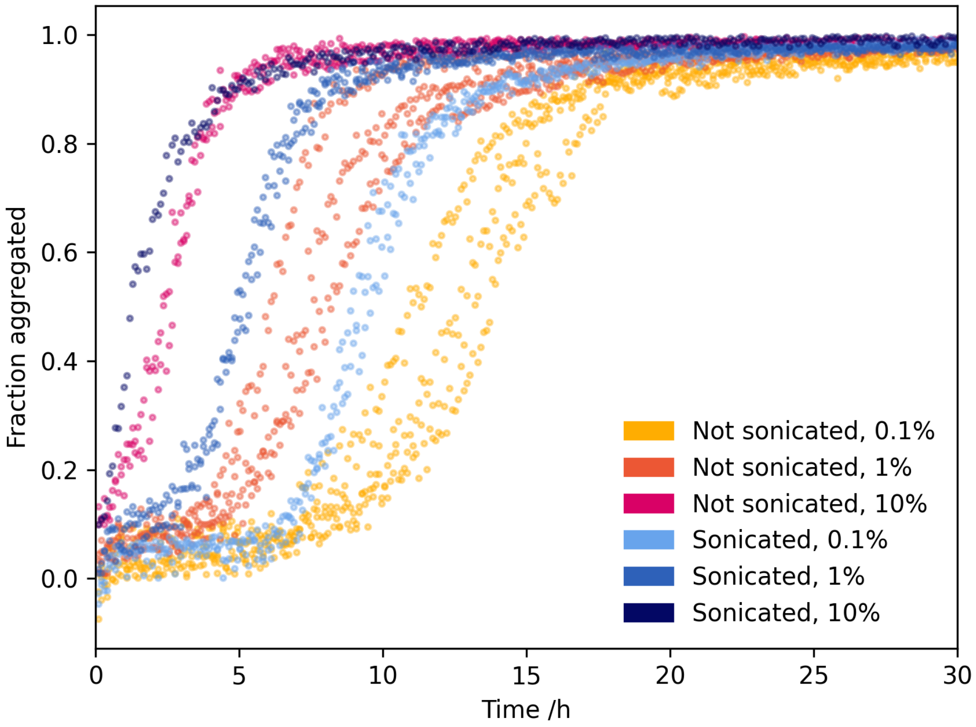

Figure S10: Sonication of fibrils increases their seeding capability. Fibrils from the same reaction were used to seed aggregation of monomers (100 µM) at a range of seed concentrations, both with and without prior sonication of the fibril seeds. This effect likely arises from the breaking up of fibril clumps, exposing further secondary nucleation sites. However, the lag time is only decreased by around 10% upon seed fibril sonication. In case sonication affected the fibril structure [2], fibrils were used as seeds without prior sonication in the experiments in this study.

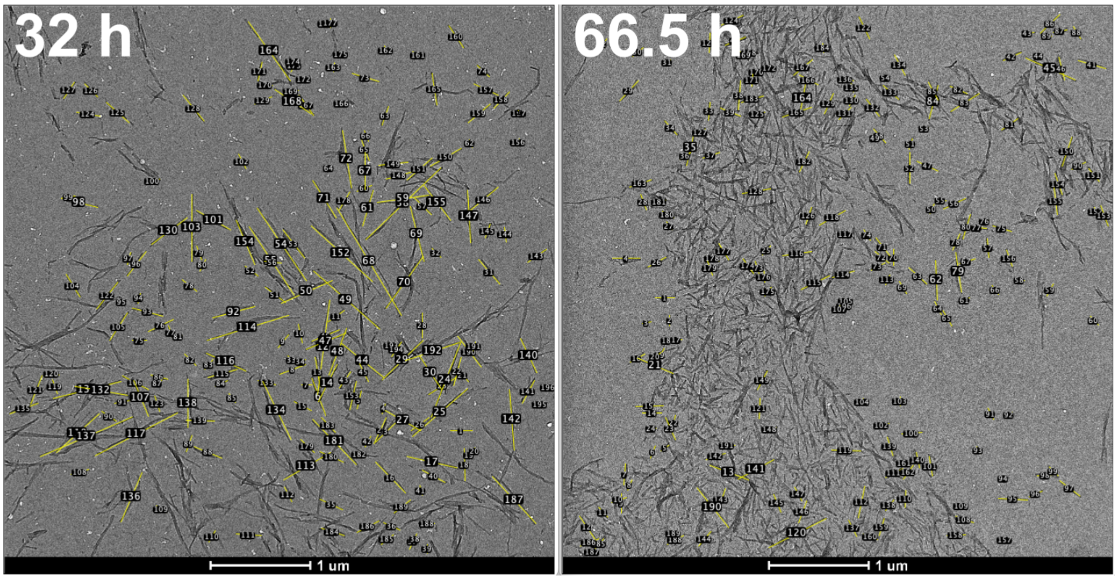

Figure S11: Example TEM images of α-synuclein fibrils after 32 (left) and 66.5 (right) hours of aggregation. The lengths of fibrils where both ends were clearly visible (yellow) were extracted using Fiji [3].

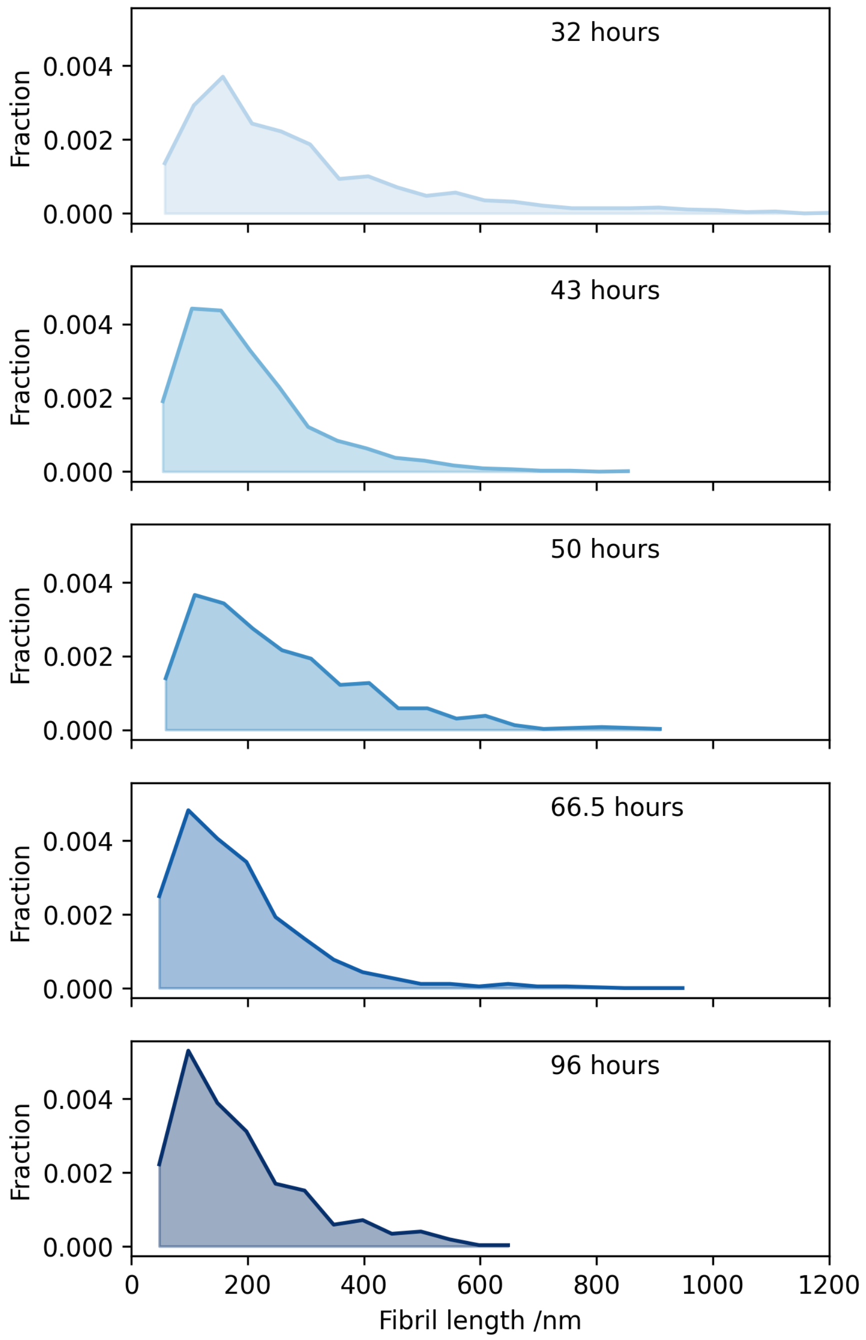

Figure S12: Length distributions of α-synuclein fibrils. Aliquots were extracted at various timepoints from an α-synuclein aggregation reaction during the plateau phase. Fibrils were imaged by TEM to determine their lengths.

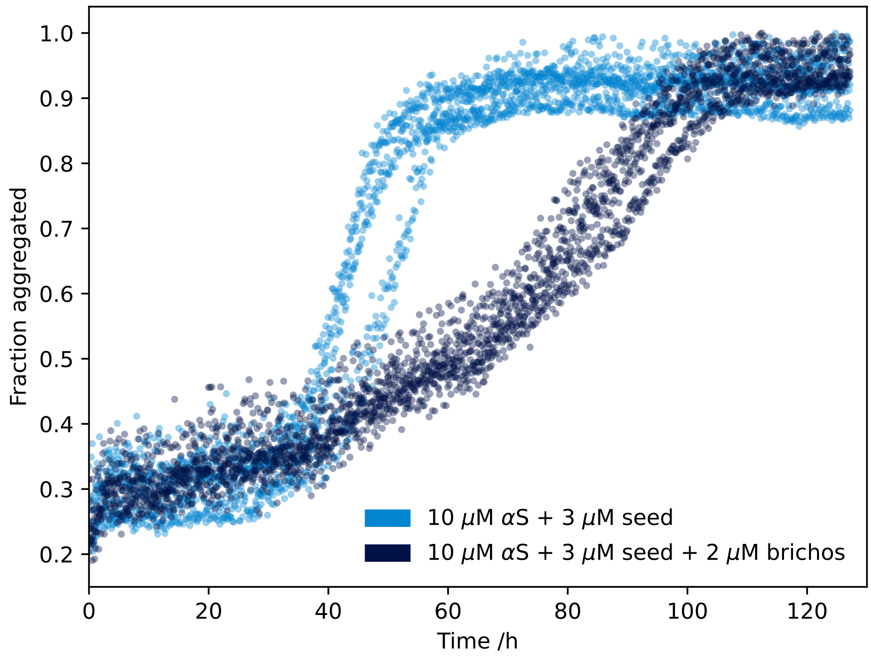

Figure S13: Brichos inhibits α-synuclein aggregation. α-Synuclein (10 μM) was aggregated in the presence of pre-formed fibril seeds both with and without brichos, in PBS with shaking (as described in Methods).

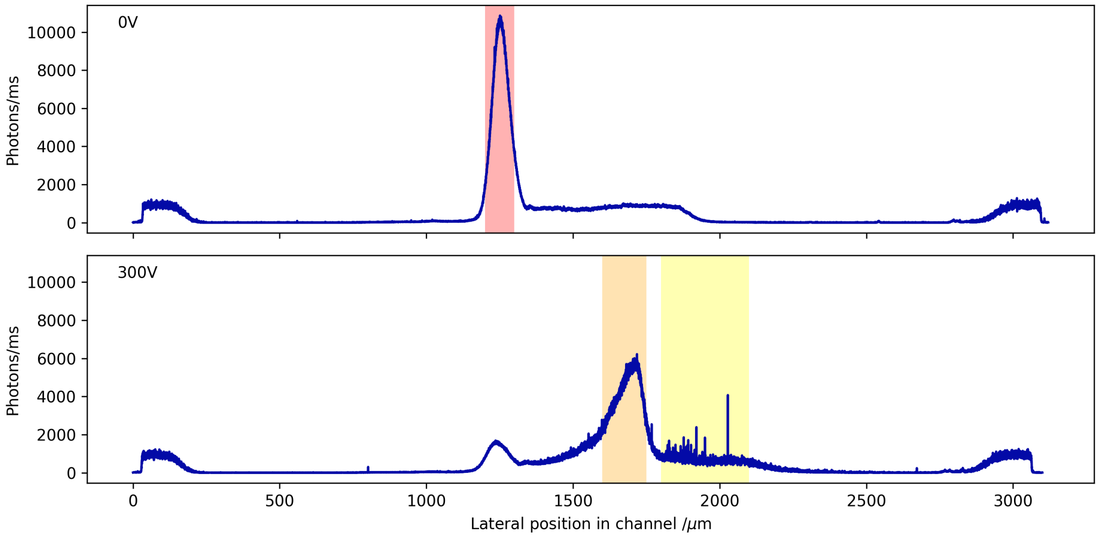

Figure S14: Oligomers have a higher mobility than monomers in the electric field. Examples of photon count rates across the channel lateral direction (parallel to the electric field). The edges of the channel are visible by the addition of Atto-488 dye to the electrolyte, evident here as the fluorescence peaks at the extremes of the channel positions. In the absence of the electric field, the whole aggregation mixture flows in a narrow stream (indicated by red). Upon the application of the 300V electric field, the sample is deflected laterally in the channel; the signal at the same position as in the 0V scan is due to protein stuck on the channel surface. Components can be separated by differences in electrophoretic mobility; the monomers (orange) are less mobile than the oligomers (yellow), due to the scaling of species charge and radius [4]. Here, only the clearly visible oligomers are highlighted (yellow) for illustrative purposes, but for our oligomer quantification we considered the whole channel width.

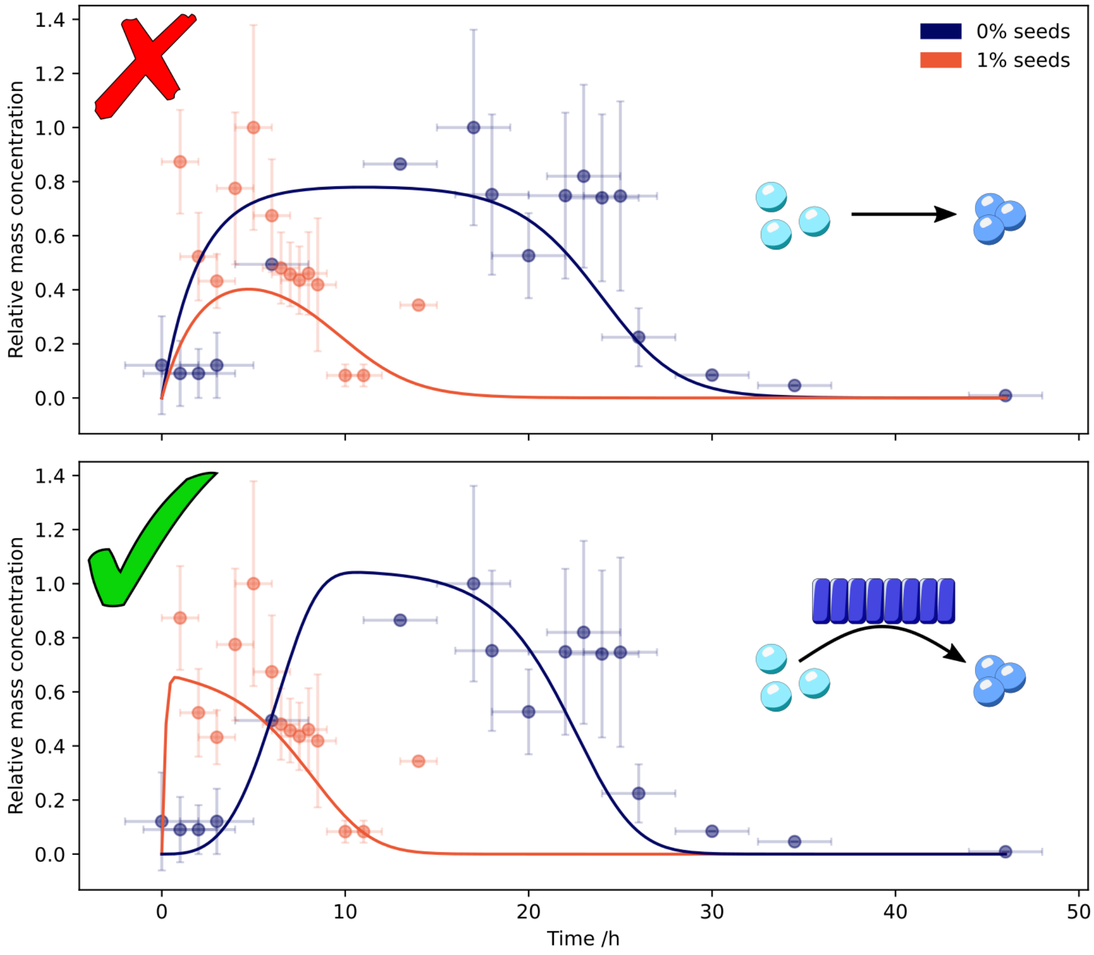

Figure S15: The main mechanism of oligomer formation is secondary nucleation. Both seeded and unseeded oligomer timecourse data during aggregation were fitted globally to a model in which oligomers only form by primary nucleation (top panel), or one including oligomer formation by secondary nucleation (lower panel). Only the model in which oligomers form by secondary nucleation is able to reproduce the experimental data.

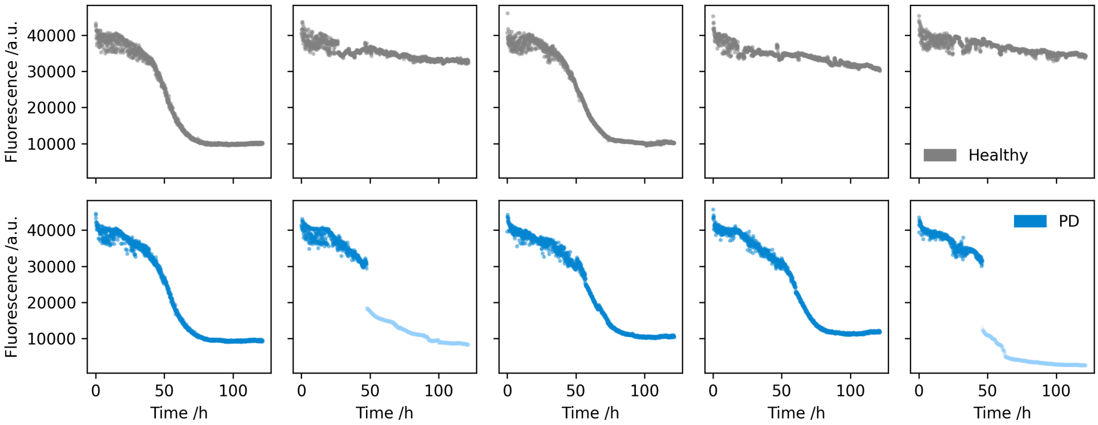

Figure S16: Aggregation kinetics of Alexa488-labelled N122C α-synuclein (100 µM) when seeded with 4% v/v pooled CSF from healthy (grey) or Parkinson's disease (blue) patients. The Parkinson's disease CSF catalysed aggregation in all five reaction wells, whereas only two of the wells with pooled healthy CSF aggregated in this time. Aggregation mixtures from two Parkinson's disease CSF-seeded wells were withdrawn (light blue) for µFFE oligomer measurements, so an accurate complete kinetic measurement could not be obtained in these cases.

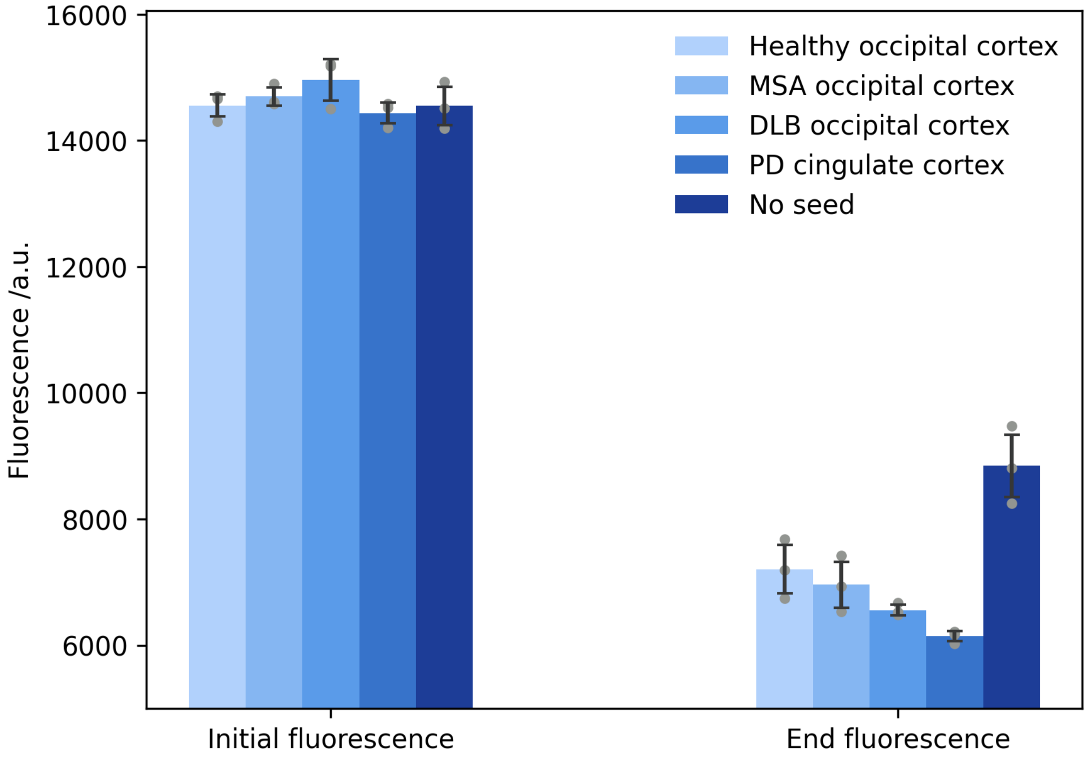

Figure S17: RT-QuIC performed using fluorescently labelled α-synuclein and brain homogenate from synucleinopathies. The decrease in fluorescence over 28 hours reflects fibril formation due to quenching of the AlexaFluor-488 dye conjugated to the α-synuclein monomers. Individual points correspond to technical replicates, bars to the mean fluorescence signal, and error bars to the standard deviation.

Supplementary tables

**Fit type**	k_n $/M^{-1}h^{-1}$	n_c	k_2 $/M^{-n_2}h^{-1}$	n_2	k_p $/M^{-1}h^{-1}$
Primary processes only (labelled α-synuclein)	2.6e-2	2	-	-	1.5e7
Fragmentation as the only secondary process (labelled α-synuclein)	1.1e-3	2	7.0e-5	1e-3	1.6e4
Both primary and secondary processes (labelled α-synuclein)	1.3e-4	2	7.4e-5	1e-3	1.2e7
Both primary and secondary processes (unlabelled α-synuclein)	1.5e-4	2	1.0e-4	1e-3	1.5e7

Table S1: Rate constants determined by fitting bulk kinetic data. k_n denotes the rate constant for the formation of nuclei from monomers, with reaction order n_c. k_2 denotes the rate constant for the fibril-dependent formation of nuclei (secondary processes), with reaction order n_2. k_p is the rate constant for fibril elongation. For the labelled α-synuclein, fits using models both including and excluding secondary processes are shown alongside the data in Figure 2, while fits for the data with k_2 fixed at the length distribution-determined fragmentation rate constant are shown in Figure 3. Fitting the AlexaFluor488-labelled N122C and unlabelled WT aggregation kinetics yielded almost identical rate constants, indicating a minimal effect of the labelling on α-synuclein aggregation kinetics.

**Fit parameter**	**Fitted value**	**Units**
k_n	1.3e-4	$M^{-1}h^{-1}$
n_c	2	-
k_2	7.4e-5	$M^{-n_2}h^{-1}$
n_2	1e-3	-
k_p	1.2e7	$M^{-1}h^{-1}$
k_{o2}	1.5e4	$M^{-n_{o2}}h^{-1}$
n_{o2}	1	-
k_{e2}	4.4e6	$M^{-1}h^{-1}$

Table S2: Rate constants determined by fitting oligomer concentrations during aggregation. Values highlighted in grey were fixed to those obtained by fitting bulk data (Table S1), while oligomer formation (k_{o2}) and dissociation (k_{e2}) rate constants were fitted according to a secondary nucleation-dominated model (for details see Methods).

References

[1] Krainer, G. *et al.* Direct digital sensing of protein biomarkers in solution. *Nature Communications* **14**, 653 (2023).

[2] Buell, A. K. *et al.* Solution conditions determine the relative importance of nucleation and growth processes in α-synuclein aggregation. *Proceedings of the National Academy of Sciences* **111**, 7671–7676 (2014).

[3] Schindelin, J. *et al.* Fiji: an open-source platform for biological-image analysis. *Nature Methods* **9**, 676–682 (2012).

[4] Arter, W. E. *et al.* Rapid structural, kinetic, and immunochemical analysis of alpha-synuclein oligomers in solution. *Nano Letters* **20**, 8163–8169 (2020).
